# Nano–Bio Interactions of Extracellular Vesicles with Gold Nanoislands for Early Cancer Diagnosis

**DOI:** 10.1155/2018/3917986

**Published:** 2018-10-09

**Authors:** S. Bathini, D. Raju, S. Badilescu, A. Kumar, R. J. Ouellette, A. Ghosh, M. Packirisamy

**Affiliations:** ^1^Optical Bio-Microsystems Laboratory, Department of Mechanical Industrial and Aerospace Engineering, Concordia University, Montreal, Canada; ^2^Atlantic Cancer Research Institute, Moncton, New Brunswick, Canada

## Abstract

Extracellular vesicles or exosomes are membrane encapsulated biological nanometric particles secreted virtually by all types of cells throughout the animal kingdom. They carry a cargo of active molecules to proximal and distal cells of the body as mechanism of physiological communication, to maintain natural homeostasis as well as pathological responses. Exosomes carry a tremendous potential for liquid biopsy and therapeutic applications. Thus, there is a global demand for simple and robust exosome isolation methods amenable to point-of-care diagnosis and quality control of therapeutic exosome manufacturing. This can be achieved by molecular profiling of the exosomes for use with specific sets of molecular-markers for diagnosis and quality control. Liquid biopsy is undoubtedly the most promising diagnosis process to advance “personalized medicine.” Currently, liquid biopsy is based on circulating cancer cells, cell free-DNA, or exosomes. Exosomes potentially provide promise for early-stage diagnostic possibility; in order to facilitate superior diagnosis and isolation of exosomes, a novel platform is developed to detect and capture them, based on localized surface plasmon resonance (LSPR) of gold nanoislands, through strong affinity between exosomes and peptide called Venceremin or Vn96. Physical modeling, based on the characteristics of the gold nanoislands and the bioentities involved in the sensing, is also developed to determine the detection capability of the platform, which is optimized experimentally at each stage. Preliminary results and modeling present a relationship between the plasmonic shift and the concentration of exosomes and, essentially, indicate possibilities for label-free early diagnosis.

## 1. Introduction

Exosomes or extracellular vesicles are vital sources of biomarkers for cancer and other pathological conditions such as inflammatory and neurodegenerative diseases and also for clinical diagnostics. They are membrane bounded nanoscale extracellular communication organelles that are released from almost all cell types to the extracellular space, transporting a cargo of active molecules (DNA, RNA, proteins/enzymes, lipid, and metabolites) to neighboring and distal parts of the body and represent real-time snapshots of the physiological/pathological status of the source cells. They are present in all body fluids, including urine, blood, ascites, and cerebrospinal fluid, and semen. When cells are cultured in laboratory or bioreactor settings, the cells discharge their exosomes in the culture-media used, called conditioned media. Exosomes are mostly spherical and their diameter ranges from 30 to 100 nm, which is about hundred times smaller than the smallest cell [1–6]. Exosomes are formed by inward budding of luminal membrane of multivesicular bodies and are constitutively released by fusion with the cytoplasmic membrane. Amongst the active molecules in the cargo, heat shock proteins (such as HSP70) are shown to be exosome membrane bound as well as inside [7–13]. It is evident in many cancers (for example, breast and ovarian) that the concentrations of the total cancer-cells and their exosome-bound HSPs are elevated [14] and implicated in various aspects of cancer biology.

The detection, isolation, and characterization of exosomes are still challenging due to the natural complexity of body fluids. For this reason, a versatile platform and an easy-to-use technique are required to adequately and selectively detect, isolate, quantify, and characterize exosomes for clinical applications. Given the growing evidence that exosomes may be the best liquid biopsy source materials for biomarker identification and discovery, there is a great demand for their simple, robust, and efficient isolation/detection from biofluids. However, currently available exosome isolation methods are precipitation based (ultracentrifugation and using polyethylene glycol) which are not suitable for point-of-care (POC) clinical-diagnosis. Ultrafiltration yields relatively pure exosomes but is technically challenging. For the development of routine exosome-based POC diagnostics, affinity-based exosome capture is technologically desirable. Most affinity-based exosome capture methods rely on monoclonal antibodies, directed against exosomes surface markers [15–18], driving higher the cost of production and inconsistency in assays such as batch-to-batch variations of antibodies. Antibody-based affinity-capture and all the other precipitation-based exosome isolation facilitate capturing all the exosomes present in the given fluid, without differentiating healthy and diseased (cancer) exosomes. Quite sophisticated micro- and nanosystems have gained attention in recent years for their high sensitivity to detect exosomes. Among them are the electrochemistry-based approaches, using electroactive molecules tagged with a detection antibody and the captured exosomes are detected by electrochemical sensing [19–21]. Nanoplasmonic sensors and microfluidic exosome analysis platforms having an antibody functionalized channel have also been reported [22, 23]. In spite of the advances in the exosome detection techniques, because of the complexity and heterogeneity of exosomes' composition, none of the existing techniques can be considered as a general method to be used for the detection of exosomes for both clinical purposes and research. The challenges are both technical and biological and any step toward solving them is useful. The development of exosome detection methods is a continuous process and there is still a lot of room for improvement.

We used a synthetic peptide (Venceremin or Vn96), having a high affinity for canonical HSPs [24, 25] as tool to capture exosomes for the platform described in this work. The rational to use Vn96 based affinity-capture of exosomes for POC diagnostic platform is that cancer cells and their exosomes over express HSPs in their lumen as well as on their surface [26], but only at a minimum level in healthy cells or their exosomes. It is also evident that cancerous cells release a higher number of exosomes, compared to normal cells implicated in tumor progression [15, 27, 28]. Thus Vn96 provide stronger affinity capture of canonical HSP-overexpressed (from cancer cells) exosomes from a given body fluid on Vn96 grafted nanoplatforms and thus may provide superior diagnostic value for early cancer diagnosis [24].

While it is important to characterize exosomes by their molecular composition, there is presently a growing demand for an accurate method to detect the absolute concentration of exosomes in body fluids for potential POC diagnosis. For this purpose, surface-based detection methods such as Surface Plasmon Resonance (SPR) and, more recently, Localized Surface Plasmon Resonance (LSPR) have emerged, in addition to flow cytometry [29], Tunable Resistive Pulse Sensing (TRPS), and Nanoparticle Tracking Analysis (NTA).

LSPR is one of the most important optical properties of gold and silver nanostructures. It occurs, when the oscillation of free conduction electrons of nanoparticles is resonant with the incident light. Both the position and the intensity of LSPR band are dependent on the size and shape of nanoparticles and they are highly sensitive to dielectric properties of the surrounding medium.

Platforms based on gold nanoislands and silver nanoislands were used for numerous sensing applications [30–36]. In this work, we have used a thermal convection method for the fabrication of nanoislands on glass substrates [33, 34]. We used a specific chemistry to covalently attach streptavidin to nanoislands, followed by grafting of the biotinylated Vn96 peptide to create a plasmonic surface where the exosomes will be captured.

The objective of the present study is to determine the detection capability of the Vn96-gold nanoislands platform by physical modeling. The density of Vn96- peptide molecules that can be accommodated on this platform has been evaluated and the concentrations of streptavidin, biotin-Vn96, and exosomes, have been estimated accordingly. At the same time, the different steps of the sensing protocol are discussed from the point of view of the successive binding events and their effect on the entire detection process. To the best of our knowledge, this is a new approach to evaluate the effectiveness of a nanoislands sensing platform, designed for the multistep, exosome capture, and detection. In an attempt to find new information that would be helpful for the exosome-based POC diagnosis, we tried to find a correlation between the experimental values of the Au-LSPR band shift and the amount of exosomes in the conditioned media of a breast cancer cell line. This modeling work is very useful in evaluating the morphology of new structures for a given size and concentration of exosomes or any other biomolecules.

## 2. Results and Discussions

### 2.1. SEM Characterization of Gold Nanoislands

**Figure 1:**
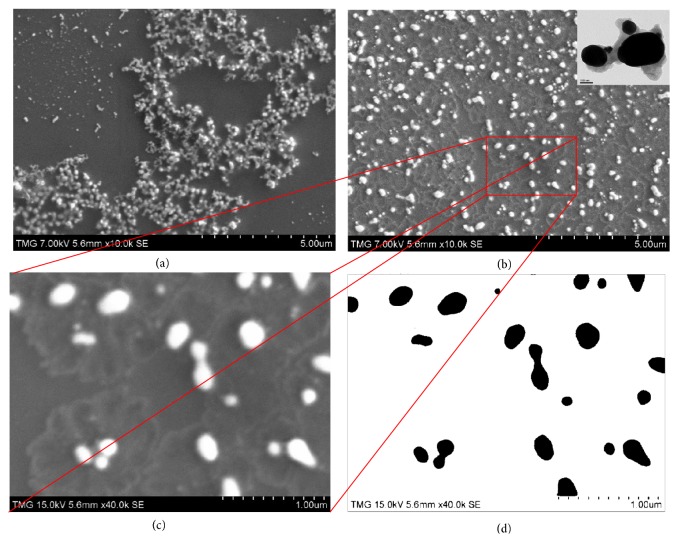
**Morphological tuning of gold aggregates to nanoislands shown by SEM images:** (**a**) SEM image of the large gold aggregates, (**b**) nanoislands (after annealing at 560°C) (inset: TEM image of nanoislands), (**c**) selected SEM image used for particle analysis, and (**d**) its binary image.

The gold nano-islands on glass substrates, formed after annealing, were characterized by Scanning Electron Microscopy (SEM) and Transmission Electron Microscopy (TEM) images. Figures 1(a) and 1(b) show the SEM images of the gold aggregates, before annealing, and that of the nanoislands obtained after annealing, respectively. From the images, it can be seen that the annealing at 560°C for 1 hour, tunes the morphology from aggregates of gold nanoparticles into nanoislands. The TEM image shows that most of the islands have an ellipsoid shape and there is a wide size distribution.

### 2.2. Image Analysis of Gold Nanoislands

The SEM image of nanoislands is analyzed using the ImageJ analysis software (Wayne Rasband, NIH, USA) to determine the dimensions of the islands and their surface density. An area of 3.2 *μ*m x 2.4 *μ*m with 20 nanoislands is considered for the analysis. The nanoislands were analyzed, and the average length and width were measured and also the average interisland distances were calculated using the center to center distance between the nearest neighbor islands. The maximum and minimum lengths of nanoislands were found to be 450 nm and 60 nm and the widths 200 nm and 60 nm. Figures 1(c) and 1(d) show the image used for the analysis and Table 1 summarizes the average values of major and minor axes of gold nanoislands, aspect ratios, interisland distances and calculated surface density.

Table 1 shows that the nanoislands are quite large, far from one to the other and their surface density is low. The size distribution of the gold nanoislands was also calculated. These calculations were based on the lower magnification SEM image shown in Fig. [Sec supplementary-material-1]. To calculate the island size distribution, an area of 12.7*μ*m x 9.52*μ*m is considered as shown in the rectangle in the Fig. [Sec supplementary-material-1]. The histogram in Fig. [Sec supplementary-material-1] clearly shows a wide size distribution (20 nm to approximately 350 nm diameter), all of the sizes having almost an equal contribution. The size distribution of nanoislands could be improved by annealing the deposited nanoparticles at the same temperature (560°C) but for a much longer time (10 h). The SEM image and the corresponding histogram are shown in Fig. [Sec supplementary-material-1] (D and F).

As the histogram shows, the sizes of the majority of islands are concentrated in the range of 20 nm to 80 nm. In spite of a better distribution, we opted for a shorter annealing time, because the size of nanoislands under these conditions is larger. Taking into account the complex multistep sensing protocol, having large nanoislands with a large penetration depth of the plasmon field would be more suitable and advantageous for higher sensitivity in the case of exosomes which are generally large in size.

### 2.3. Physical Modeling

In this section, we have built up a model based on the quantitative analysis of the nanoislands shown in the previous section. Taking into account the average size and the surface density of nanoislands as found from the SEM data, we have calculated the number of the different entities involved in the bio-sensing process that can be immobilized on the surface of nanoislands. When immobilizing the successive layers on the surface of a nanoisland, we have considered their dimensional sizes as known from the literature [37, 38]. A typical gold nanoisland has the shape of an ellipsoid as shown in Fig. [Sec supplementary-material-1] with average dimensions of 200 nm (L), 150 nm (W) and 50 nm (T) obtained from the particle analysis. The maximum area available for immobilization with these ellipsoidal nanoislands is approximated as

(1) $$\begin{array}{c} A\approx 4\pi {\left(\;\frac{{{{\left(LW\right)}^{1.6}+\left(LT\right)}^{1.6}+\left(WT\right)}^{1.6}}{3}\;\right)}^{1/1.6} \end{array}$$

The surface area of nanoislands limits the number of different ligands that can be immobilized on its surface. The surface area calculated using (1) was used for further evaluations. Fig. [Sec supplementary-material-1] shows that, in spite of the larger size of the bio entities involved in the sensing process, the decaying plasmon field of a large nanoisland reaches the exosomes that are the target of the detection process. Fig. [Sec supplementary-material-1] shows the cross section of one nanoisland, carrying the successive layers of compounds involved in the detection of exosomes. As shown in the model (Fig. [Sec supplementary-material-1]), the ligands consist of a linker and the streptavidin bound to biotin-Vn96. The shape of the linker-streptavidin-biotin complex is assumed to be a rectangular body with the dimensions of 4.2 nm x 4.2 nm x 9 nm [37, 38]. The next layer consists of Vn96, a polypeptide specifically designed to capture exosomes, having a predictive spherical shape with a diameter of 2 nm and the exosomes are modeled as spheres with a diameter of 100 nm. Taking into account the available surface area, the numbers of Vn96 and exosomes that can be accommodated per nanoisland were estimated based on their physical dimensions and constraints and shown in Table 2.

It can be seen from the Table 2 that each nanoisland can accommodate a maximum of 1976 ligand of streptavidin, 12810 Vn96 molecules and only 9 exosomes. In our experiments, each Vn96 molecule is linked with a biotin through a PEG linker; therefore, the number of Vn96 a nanoisland can accommodate is equal to the number of biotin complexes, that is, 12810 on each nanoisland. As streptavidin can bind to 4 biotin molecules, while only 7904 biotin-PEG-Vn96 molecules can be bound to the maximum available streptavidin and the rest of the molecules will be washed away.

Theoretically, each Vn96 molecule can latch onto the exosome by binding to one heat shock protein contained on the surface of the exosomes. From the physical modeling, it is clear that the number of Vn96 molecules available to capture the exosomes is much higher than the exosomes that each nanoisland can accommodate. Hexagonal orientation of exosomes is considered, in order to understand how many Vn96 molecules could actually contribute to capture the exosomes. The percentage of Vn96 molecules involved in bonding of exosomes is very low, only around 5%. Fig. [Sec supplementary-material-1] (D and E) show the orientation of exosomes, while Fig. [Sec supplementary-material-1] shows the top view and Fig. [Sec supplementary-material-1], the isometric view of the exosomes captured by the Vn96 molecules. The Vn96 molecules which actually contribute to the capture of exosomes are shown in orange as seen in Fig. [Sec supplementary-material-1] (D and E).

The biosensing protocol, tailored to detect the exosomes, consists of immobilization of different compounds on gold nanoislands, by binding or by adsorption of layers with varying thicknesses. For the approach used here, it is important to optimize the two step: the streptavidin-biotin binding and the capture of exosomes, while the concentrations of Nanothink and EDC-NHS used here are similar to those used for biosensing of other biomolecules [39]. For this purpose, the concentration and the amount of the entities involved in biosensing protocol were varied, to maximize the shift of the Au plasmon band, at each of the important steps. As the main step in deciding the outcome of the sensing process is the Vn96-exosome interaction, it seemed reasonable to optimize the formation of the streptavidin, biotin complex, in the specific case of biotin, connected to Vn96, through a PEG moiety. Because of the high affinity and the stability, the streptavidin-biotin model system and its structure and mechanism of formation have been thoroughly investigated [40–42]. This is not the case for the biotin-PEG-Vn96 complex, synthesized quite recently, with the only objective of binding the exosomes.

**Figure 2:**
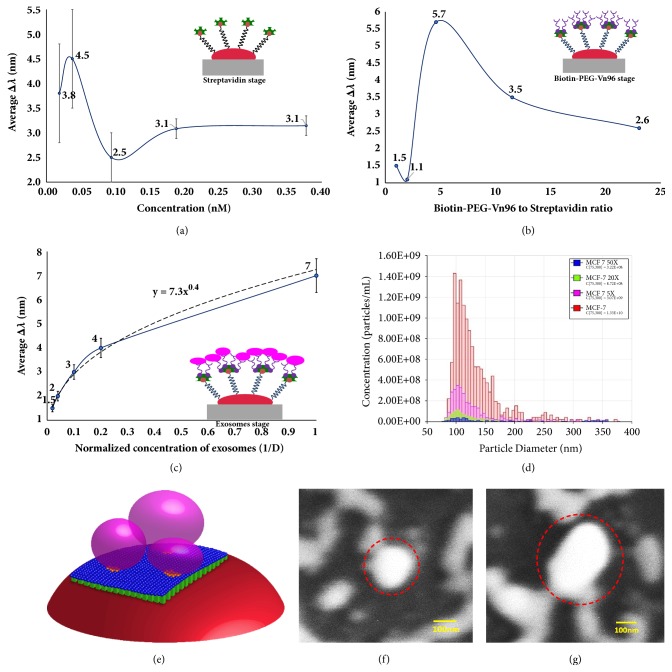
**Plasmonic shift due to streptavidin and Biotin-PEG-Vn96 interactions.** (**a**) Dependency of ∆*λ* on the concentration of streptavidin only. (**b**) Ratio of Biotin-PEG-Vn96 to Streptavidin. (**c**) Dependency of the shift of Au LSPR band on the concentration of MCF-7 exosomes. (**d**) Size distribution of MCF7 exosomes as obtained by Tunable Resistive Pulse Sensing (TRPS) measurements. (**e**) Exosomes with different sizes and shapes captured by Vn96 molecules. (**f, g**) SEM images of exosomes captured by gold nanoislands during the last step of the biosensing (exosomes are marked in circles).

First, only the concentration of streptavidin solution was varied to find the concentration that will result in the maximal shift of the Au LSPR band. The dependency of ∆*λ* on the concentrations of streptavidin is shown in Figure 2(a). The highest shift (4.5 nm) is seen when the concentration of streptavidin is around 0.04 nM. From the experimental results, it has been observed that, at lower concentrations of streptavidin, the LSPR shift values are unstable, compared to those corresponding to higher concentrations. Therefore, instead of considering concentrations with the highest LSPR shift, the concentration with a more stable LSPR shift is considered for further optimization of the biosensing protocol. The streptavidin concentration of 0.19 nM is considered and the concentration of Biotin-PEG-Vn96 is varied to study the effect of the ratio of Biotin-PEG-Vn96 to streptavidin. Figure 2(b) shows the dependency of the average LSPR shift when different ratios of biotin-PEG-Vn96 to streptavidin are used, keeping streptavidin concentration constant at 0.19 nM.

It is wellknown that one streptavidin molecule can bind to four biotin molecules and the LSPR shift corresponding to this ratio is maximal [40, 43]. Hence, if the ratio of streptavidin to biotin is maintained around four, the LSPR shift of Au has to be the largest. It is noticeable from Figure 2(b) that, as expected, the maximum shift is obtained when the ratio of biotin to streptavidin is maintained around four. This corresponds to 5.23 x10^14^ biotin-PEG-Vn96 complexes containing 1.14 x10^14^ molecules of streptavidin. Further, the LSPR shift decreases with the increase in ratio of the complex. The results have shown that, working with lower concentrations of biotin-PEG-Vn96 to streptavidin, the LSPR shift values show a poor repeatability. This might be because of less available biotin-PEG-Vn96 molecules, compared to those that may be accommodated by the streptavidin molecules and, in this situation, the binding is random. Comparing this number with the one calculated theoretically, it can be seen that, under the conditions of the experiment, a considerably higher number of complexes were formed. However, if we consider the removal of the nonbound complexes through the washing process, the number of molecules from the experimental results matches those from the physical modeling, validating the model.

The results show that the surface density of nanoislands, fabricated through the thermal convection method, is high enough to accommodate the biotin-PEG-Vn96 complex molecules, involved in the experiment. The optimized concentrations of all the entities used in the biosensing protocol and the corresponding LSPR shifts are summarized in [Sec supplementary-material-1]. The amount (volume) of each entity in the biosensing protocol is chosen to cover the whole sensing area.

The dependency curve, showing the shift of the Au LSPR band for different concentrations of exosomes, namely, corresponding to dilution factors of 50x, 25x, 10x, 5x, 1x is shown in Figure 2(c). Figure 2(d) represents the size distribution and counts of exosomes corresponding to different dilutions of MCF7 exosomes, quantified using the Tunable Resistive Pulse Sensing (TRPS) (qNano from iZON science) instrument. The dependency curve (Figure 2(c)) is built by using the concentration of MCF-7 exosomes, containing 1.33 X 10^10^ particles/ml. From this plot, it can be seen that the average shift increases as the dilution factor decreases and reaches the highest shift for the undiluted sample. It has to be noted that the equation of the curve reflects a nonlinear trend in the form of Lx^M^ where x corresponds to the number of exosomes-Vn96 interactions. It can be observed from the experimental results that the LSPR shift has a nonlinear dependency on binding to the Vn96 and that the curve is not saturated, which clearly shows that the capacity of the nanoisland platform is still high enough to capture more exosomes than the actual number of exosomes in the culture media used in this experiment.

For modeling purposes, exosomes were considered spherical and having a uniform size (100 nm) but, actually, their size varies from 30 to 100 nm and the shape may not be exactly spherical as shown in Figure 2(e). An exosome, captured by a gold nanoisland is shown in Figure 2(f), whereas Figure 2(g) shows a cluster of exosomes, captured by a larger gold nanoisland as depicted in the modeling. This can be clearly seen in the in Fig. [Sec supplementary-material-1] (A and B). The number of available HSP on the surface of exosomes varies depending on the size of exosomes and the stage of the disease. When exosomes are captured by Vn96, the interactions could result in a deformation due their elastic nature. Hence, the binding of HSP to the Vn96 molecules (protein–protein binding) will result in a nonlinear behavior. The dependency curve allows the estimation of the concentration in terms of the number of exosomes in the sample.

**Figure 3:**
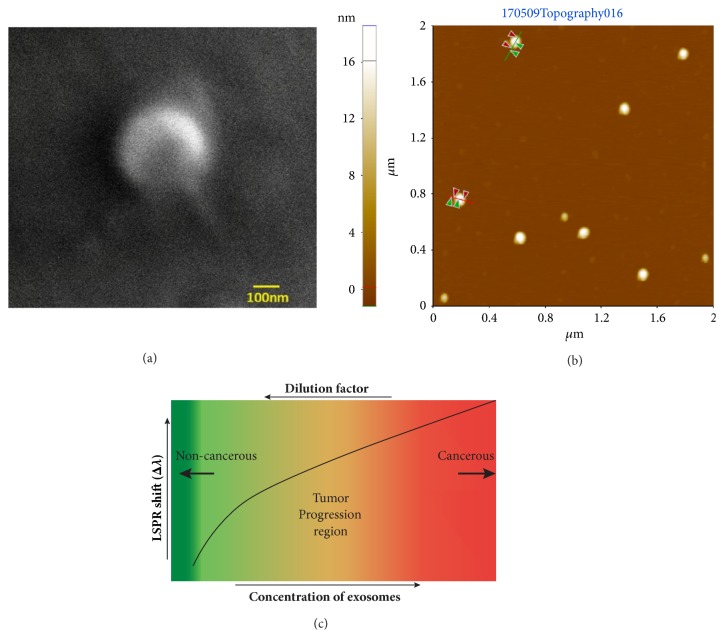
**SEM, AFM images of exosomes and the relationship between the plasmonic shift and concentration.** (**a**) SEM image of the MCF7 exosomes. (**b**) AFM image of the MCF7 exosomes. (**c**) The relationship between plasmonic shift and concentration of exosomes during cancer progression.

Particle analysis has shown that the density of nanoislands is around 3 nanoislands/*μ*m^2^. As found by modeling, each of them can accommodate 9 exosomes, that is, a total of 27 exosomes in an area of 1 *μ*m^2^ of the developed platform. A previous study has indicated that the average number of exosomes that can be captured from a glioblastoma patient's plasma on anti-CD63 monoclonal-antibody grafted 2D planner surface of the chip is 9 microvesicles/*μ*m^2^ [15], whereas our platform is able to accommodate a much higher number of exosomes. As the sample used in this work corresponds to the cell culture of a breast cancer cell-line, the concentration of exosomes in the undiluted sample may be attributed to a cancerous condition as shown in Figure 3(c), where the total number of exosomes in the unit volume of plasma is noticeably higher than in the normal plasma [15]. Alternatively, the concentration of exosomes corresponding to a 50x dilution would mean a noncancerous situation or a very-early stage of the disease. Hence, the tested range of concentrations covers a wide range, starting with concentrations of exosome present in a given body-fluid, simulating a noncancerous, early-stage disease, to a fully developed cancerous condition. Thus, one can infer that the nanoisland platform developed in this work, based on LSPR, can effectively detect from early stage to advanced stages of cancer.

SEM and AFM images of exosomes from the breast cancer cells (MCF7) are shown in Figures 3(a) and 3(b) respectively. The relationship between the LSPR shift and the concentration of exosomes during cancer progression is schematically shown in Figure 3(c). It is known that the concentration of exosomes present in the body fluids increases tremendously (by many folds) in a cancer patient, compared to a healthy patient. For example, in the case of a glioblastoma patient's plasma study mentioned above [15], the concentration of exosomes was found to be approximately 50 times higher than that corresponding to a healthy patient. Hence, we could envisage that the concentration of exosomes, detected by measuring the LSPR plasmonic shift, will reflect the progression of cancer as shown in Figure 3(c).

## 3. Conclusion

In this work, the sensing protocol for the LSPR detection of extracellular vesicles, based on their high affinity to the Vn96 polypeptide was optimized. A simple physical model was developed by analyzing the characteristics of gold nanoislands, calculating their surface area and the number of the different species that can be successively immobilized on the surface of a nanoisland. It is estimated that the most important step in the protocol, deciding the outcome of the overall sensing process, is the formation of the streptavidin-biotin-PEG-Vn96 complex. Therefore, the concentrations of streptavidin and biotin-PEG-Vn96 complex were optimized experimentally and the maximum LSPR shift was found for the ratio of 1 to 4 in agreement with the physical modeling. Particle analysis performed on SEM images has shown that the density of nanoislands is around 3 nanoislands/*μ*m^2^. By modeling, it was found that each of the nanoislands can accommodate 9 exosomes, that is, a total of 27 exosomes per *μ*m^2^. Practically, it means that the developed Au nanoisland platform can capture a much higher number of extracellular vesicles than that present in the MCF7 sample used for this study, providing a very broad detection range covering from early stages to advanced stages. Based on our preliminary results, the novel LSPR detection method of extracellular vesicles could be used as a tool to diagnose cancer at an early stage of the disease.

## 4. Materials and Methods

### 4.1. Materials

The substrates used in this experiment are the microscope glass slides purchased from Technologist Choice, Bio Nuclear diagnostics Inc., with a glass transition temperature, T_g_ = 820°C. Substrates are cut to the size of 37 mm x 12.5mm x 1 mm. Gold (III) chloride trihydrate (HAuCl_4_.3H_2_O) and sodium citrate were purchased from Sigma Aldrich. De-ionized (DI) water with a resistivity of 18MΩ, used in all the experiments was obtained from the NANO pure ultrapure water system (Barnstead). 11-mercaptoundecanoic acid in ethanol (Nano Thinks Acid 11), N-(3-Dimethylaminopropyl)-N′-ethylcarbodiimide hydrochloride (EDC) and N-Hydroxy succinimide (NHS), and phosphate buffered saline (PBS) was obtained from Sigma Aldrich, Canada. PBS tablets were dissolved in DI water at 0.1M concentration (pH = 7.2). Streptavidin was purchased from IBA GmBH. Biotin-PEG-Vn96 was purchased from New England Peptide. MCF7 exosomes were purified from MCF7 cells conditioned media in a bioreactor, using ultracentrifugation, and resuspended in fetal bovine serum free cell-culture media and the exosome concentration was determined by using NTA [24].

### 4.2. Fabrication of the Gold Nano-Island Platform

Three-dimensional (3D) gold (Au) nanoisland structures on glass substrates were fabricated from gold colloidal solution that, subsequently, was deposited on glass substrates by the thermal convection method. The gold colloidal solution was prepared by the reduction of Gold (III) Chloride trihydrate (HAuCl_4_.3H_2_O) (chloroauric acid) by sodium citrate, following Turkevich's method [44]. Briefly, 15 mg of HAuCl_4_.3H_2_O was dissolved in 90 mL of DI water and heated until the solution reached its boiling point. Then, 5 mL of 2% sodium citrate solution is added to the boiling solution. After the addition of sodium citrate, a change in color from the original yellow to a transparent purple (wine red) can be clearly observed, showing the presence of gold nanoparticles. Meanwhile, the glass substrates were cleaned with soap solution and DI water, then rinsed with acetone, 2-propanol (IPA), and dried. Further, the substrates were heated at 100°C in an oven for 1 hour in order to remove the moisture and possible contaminants. The glass substrates were immersed in the beaker containing the gold colloidal solution at an angle of approximately 30^0^ and kept at a temperature between 50 and 55°C. The Au nanoparticles from the colloidal suspension will slowly evaporate and deposit multilayers of Au particles on the substrate as shown in Fig. [Sec supplementary-material-1] (A and B). Au nanoislands were fabricated by annealing the samples at 560°C for one hour and 10 hours.

### 4.3. Biosensing Protocol

The different steps involved in the biosensing protocol and their corresponding LSPR shifts are shown in Fig. [Sec supplementary-material-1] (A and B). The first step consists of the functionalization of the Au nanoparticles by immersing the sample into the solution of 11-mercaptoundecanoic acid (Nanothink) (5mM) for 3 hours and then allowing it to dry. After this step, the absorption spectrum is measured using the Perkin Elmer spectrophotometer (Lambda650). The shift of the Au plasmon band towards longer wavelengths confirms the presence of the self-assembled monolayer of linker molecules on the surface of nano-islands. In the next step, the monolayer formed on the surface of Au nanoislands is activated by adding 200 *μ*L of cross-linker, which is a mixture (1:1) of 0.1M N-(3-dimethylaminopropyl)-N′-ethylcarbodiimide hydrochloride (EDC) and 0.05M N-Hydroxysuccinimide (NHS), and then incubated for 2 hours. After drying, the spectrum is measured again. The next step is the immobilization of streptavidin to the activated linker layer. 200 *μ*L of a 0.19 nM solution of streptavidin in water is added to the activated linker layer and incubated for an hour and left to dry before taking a spectral measurement. In the next step, 200 *μ*L of a 0.87 nM of biotin-PEG-Vn96 solution is deposited on top of the streptavidin layer and incubated for 4 hours and dried and the spectral measurement is taken again. The final step of the biosensing protocol includes in the deposition of 200 *μ*L of the MCF 7 (breast cancer cell line) cell culture conditioned media, containing EVs/exosomes for their relative immobilization.

## Figures and Tables

**Table 1:** Average physical characteristics of the gold nanoislands prepared by thermal convection.

Length	198.89 nm

Width	146.67 nm

Aspect ratio	1.37

Inter-island distance	342.5 nm

Surface density	3 nano-islands/*μ*m^2^

**Table 2:** Surface area of a single nanoisland and the maximal number of ligands that can be accommodated.

**Streptavidin**		**Biotin-PEG-Vn96**		**Exosomes**	
*Surface area of nano-island (A1)*	*No. of ligands*	*Surface area for Vn96 (A3)*	*No. of Vn96*	*Surface area for exosomes (A4)*	*No. of exosomes *

3.486 x10^4^ nm^2^	1976	4.49 x10^4^ nm^2^	12810	1.16 x10^5^ nm^2^	9

## Supplementary Materials

Supplementary Materials
* Fig.S1: Size distributions of gold nanoislands under two different annealing times.* (A) Large area SEM image of gold nanoislands after 1-hour annealing; (B) binary image of 1-hour annealed gold nanoislands; (C) histogram corresponding to size distribution of 1-hour annealed gold nanoislands; (D) SEM image of gold nanoislands after 10 hours of annealing; (E) binary image of 10 hours annealed gold nanoislands; (F) histogram corresponding to size distribution of 10 hours annealed gold nanoislands.* Fig.S2: Physical modeling of interactions.* (A) Gold nanoisland in the shape of a half-ellipsoid (B) decay of the plasmon field of a large nanoisland. (C) Cross section of one nanoisland immobilized with the successive layers of bioentities involved in the detection of exosomes. Exosomes captured by Vn96 molecules ((D) top view and (E) isometric view). The spheres in pink and blue represent the exosomes and the Vn96 molecules, respectively.* Fig.S3: SEM image of gold nanoislands with and without exosomes.* (A) SEM image of the gold nanoislands without exosomes. (B) SEM image of the gold nanoislands with exosomes.* Fig.S4: Fabrication of gold nanoisland platforms.* (A) Schematic of the convective assembly process. (B) Schematic of the morphological tuning of gold multilayers to gold islands by annealing.* Fig.S5: Biosensing protocol and their corresponding absorbance bands.* (A) Schematic of the biosensing protocol. (B) Au nanoisland plasmon band corresponding to the different steps of the protocol.* Table S1: Concentrations and volume of the entities used in the biosensing protocol with their corresponding average LSPR shift*.
