# Supplementary material for: Nano–Bio Interactions of Extracellular Vesicles with Gold Nanoislands for Early Cancer Diagnosis
Source: Research (Wash D C). 2018 Oct 9;2018:3917986. doi: 10.1155/2018/3917986 (PMC6750071; doi:10.1155/2018/3917986)
Supplement: Supplementary Materials — Fig.S1: Size distributions of gold nanoislands under two different annealing times. (A) Large area SEM image of gold nanoislands after 1-hour annealing; (B) binary image of 1-hour annealed gold nanoislands; (C) histogram corresponding to size distribution of 1-hour annealed gold nanoislands; (D) SEM image of gold nanoislands after 10 hours of annealing; (E) binary image of 10 hours annealed gold nanoislands; (F) histogram corresponding to size distribution of 10 hours annealed gold nanoislands. Fig.S2: Physical modeling of interactions. (A) Gold nanoisland in the shape of a half-ellipsoid (B) decay of the plasmon field of a large nanoisland. (C) Cross section of one nanoisland immobilized with the successive layers of bioentities involved in the detection of exosomes. Exosomes captured by Vn96 molecules ((D) top view and (E) isometric view). The spheres in pink and blue represent the exosomes and the Vn96 molecules, respectively. Fig.S3: SEM image of gold nanoislands with and without exosomes. (A) SEM image of the gold nanoislands without exosomes. (B) SEM image of the gold nanoislands with exosomes. Fig.S4: Fabrication of gold nanoisland platforms. (A) Schematic of the convective assembly process. (B) Schematic of the morphological tuning of gold multilayers to gold islands by annealing. Fig.S5: Biosensing protocol and their corresponding absorbance bands. (A) Schematic of the biosensing protocol. (B) Au nanoisland plasmon band corresponding to the different steps of the protocol. Table S1: Concentrations and volume of the entities used in the biosensing protocol with their corresponding average LSPR shift. [file 3917986.f1.docx]

**Supplementary Materials:**


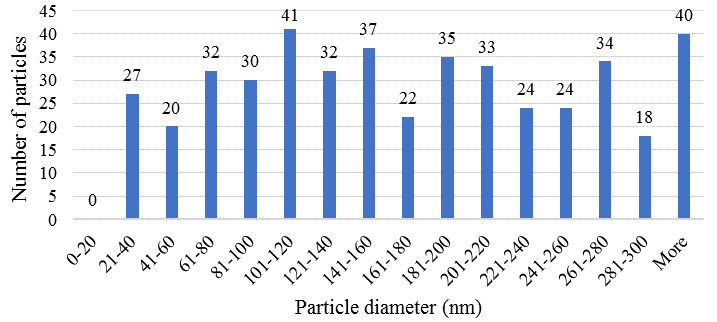


C


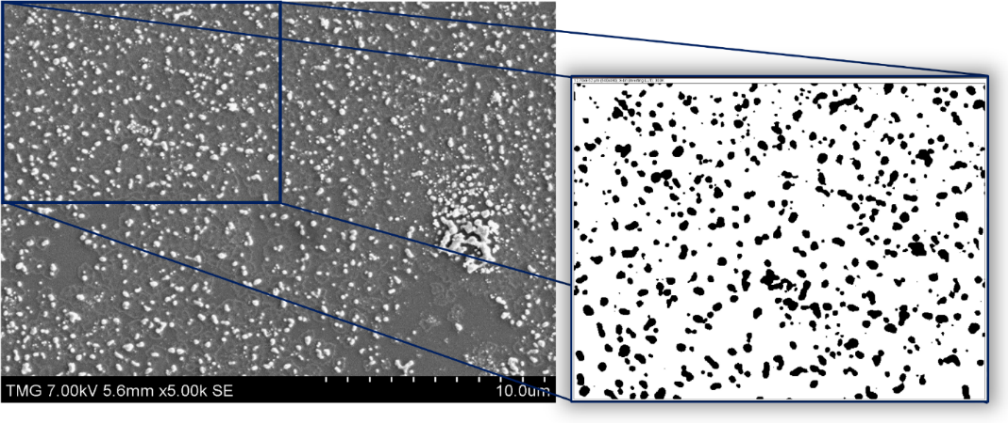


B

A

D


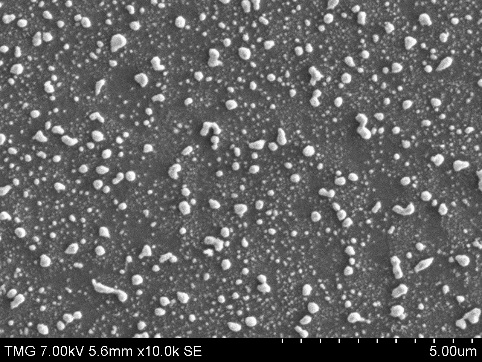


E


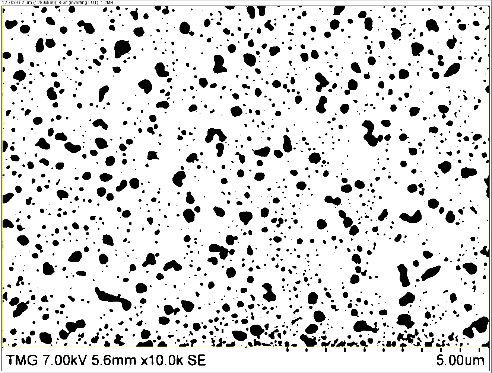


F


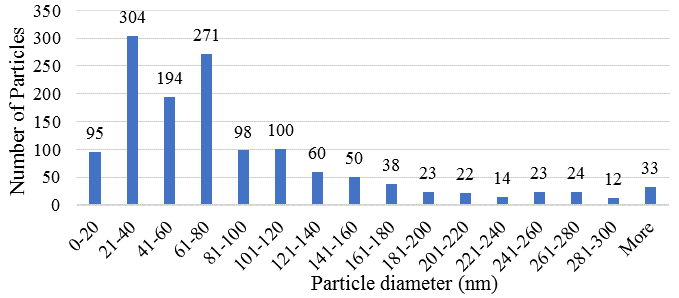


**Fig.S1. Size distributions of gold nano-islands under two different annealing times** (**A**) Large area SEM image of gold nano-islands after 1-hour annealing (**B**) its binary image (**C**) and the histogram corresponding to their size distribution; (**D**) after 10 hours annealing (**E**) binary image (**F**) and the histogram corresponding to their size distribution


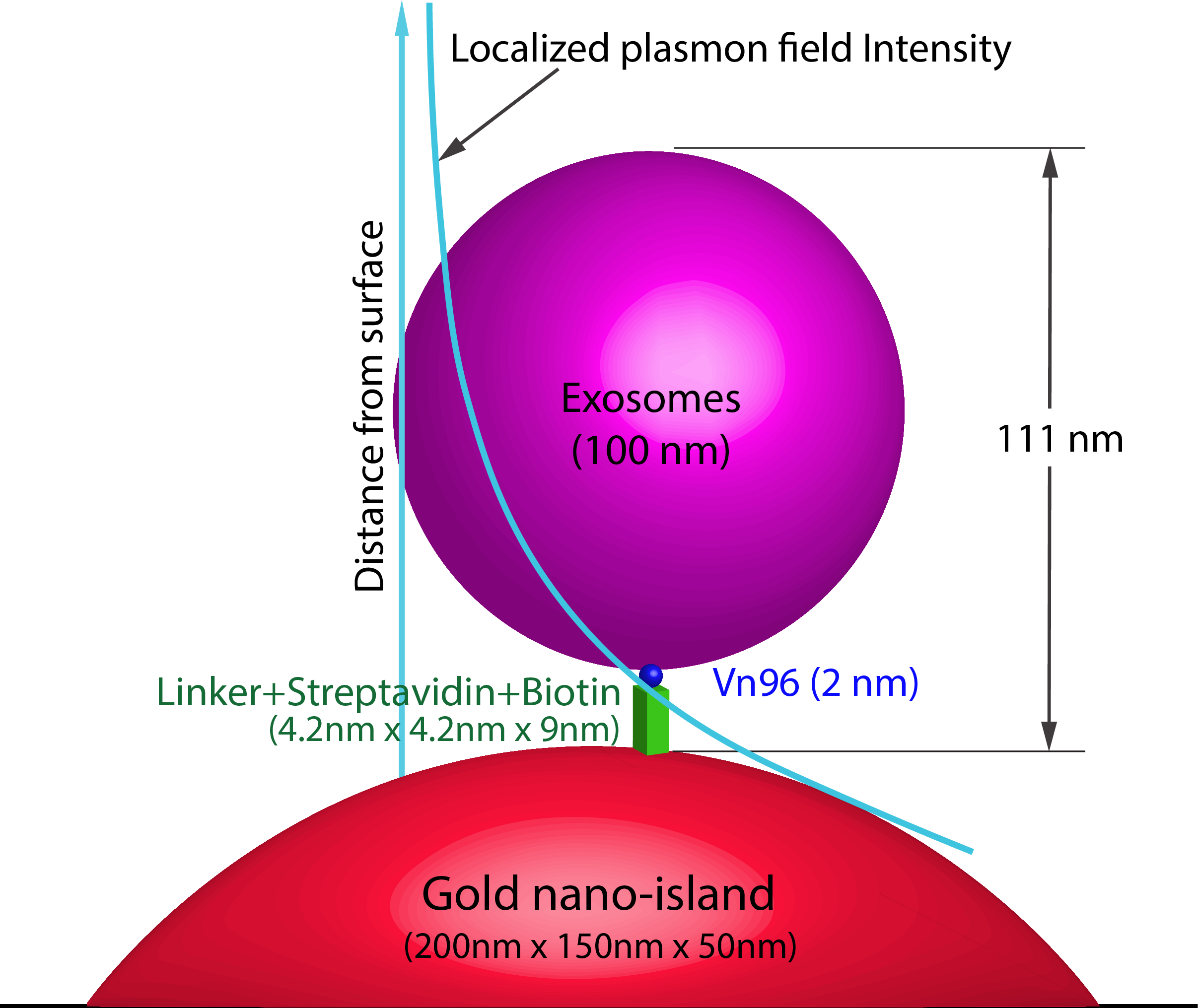


B

A


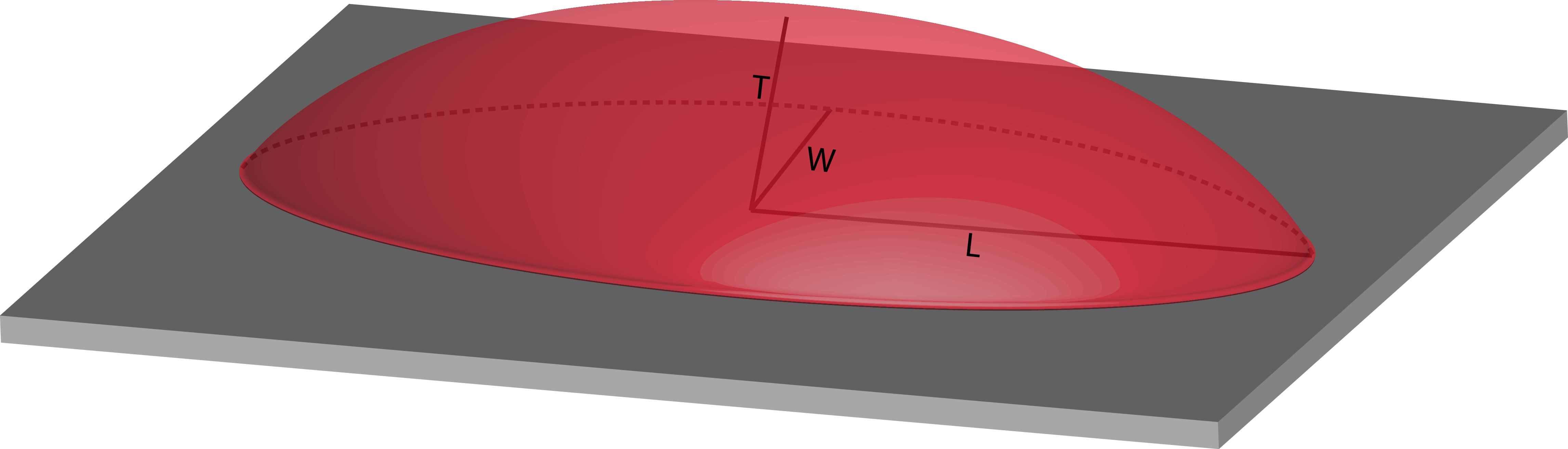


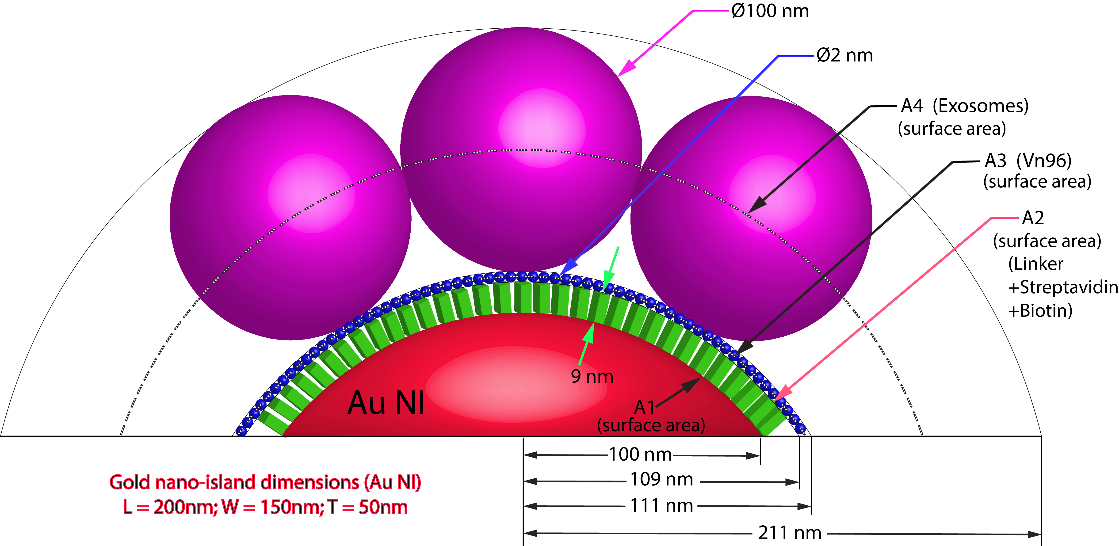


C

E


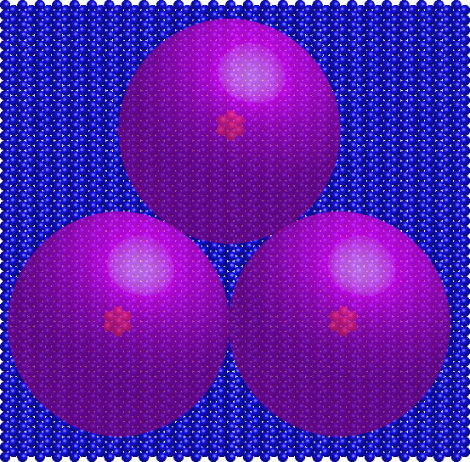

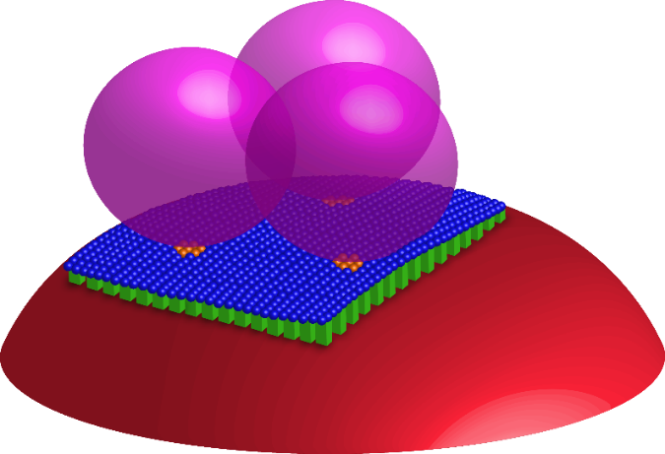


D

**Fig.S2. Physical modeling of interactions** (**A**) Gold nano-island in the shape of a half-ellipsoid (**B**) decay of the plasmon field of a large nano-island (**C**) Cross-section of one nano-island immobilized with the successive layers of bio-entities involved in the detection of exosomes Exosomes captured by Vn96 molecules ((**D**) top view and (**E**) isometric view). The spheres in pink and blue represent the exosomes and the Vn96 molecules, respectively

**Fig.S3. SEM image of gold nano-islands with and without exosomes** (**A**) SEM image of the gold nano-islands without exosomes (**B**) SEM image of the gold nano-islands with exosomes


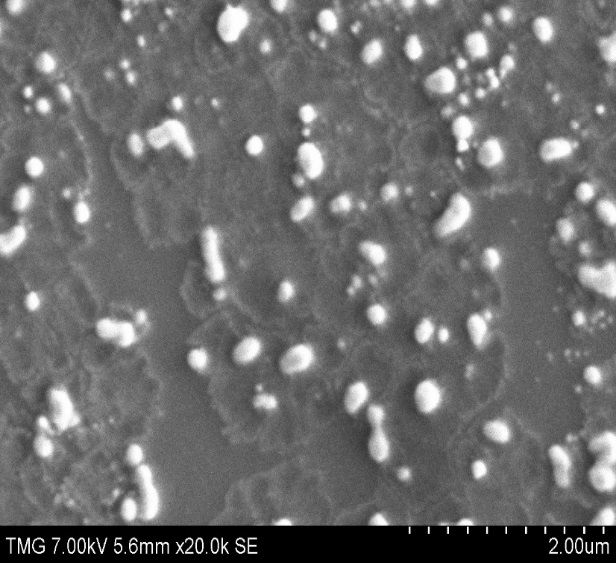

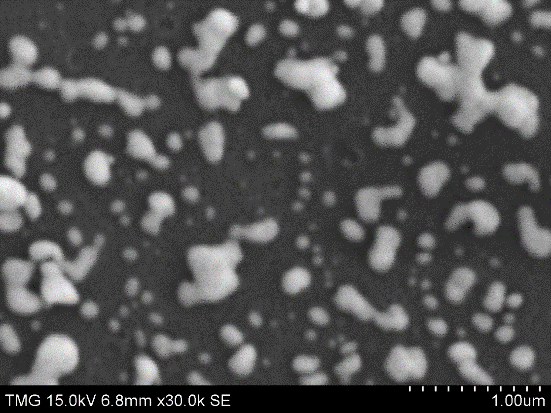


**A**

**B**


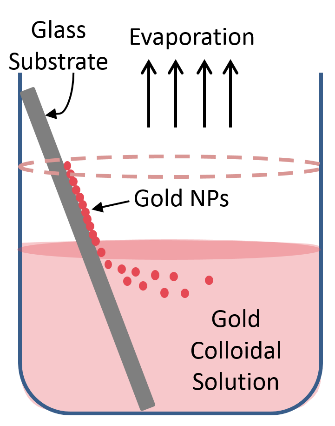

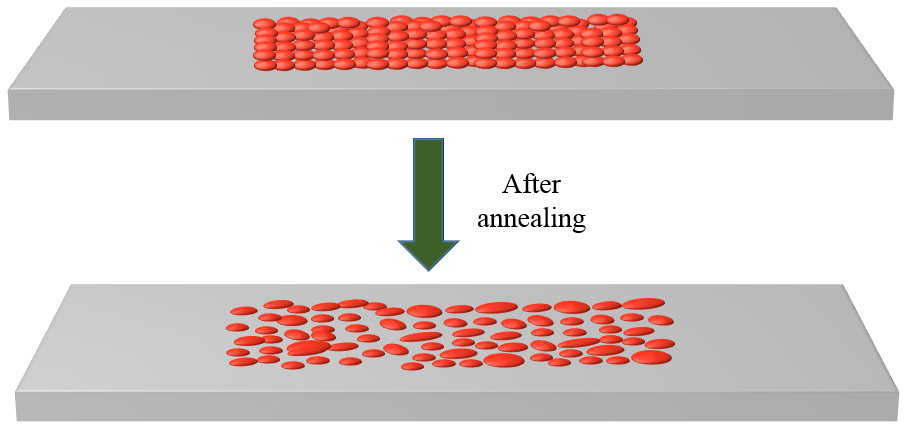


A

B

**Fig.S4. Fabrication of gold nano-island platforms** (**A**) schematic of the convective assembly process (**B**) schematic of the morphological tuning of gold multilayers to gold islands by annealing


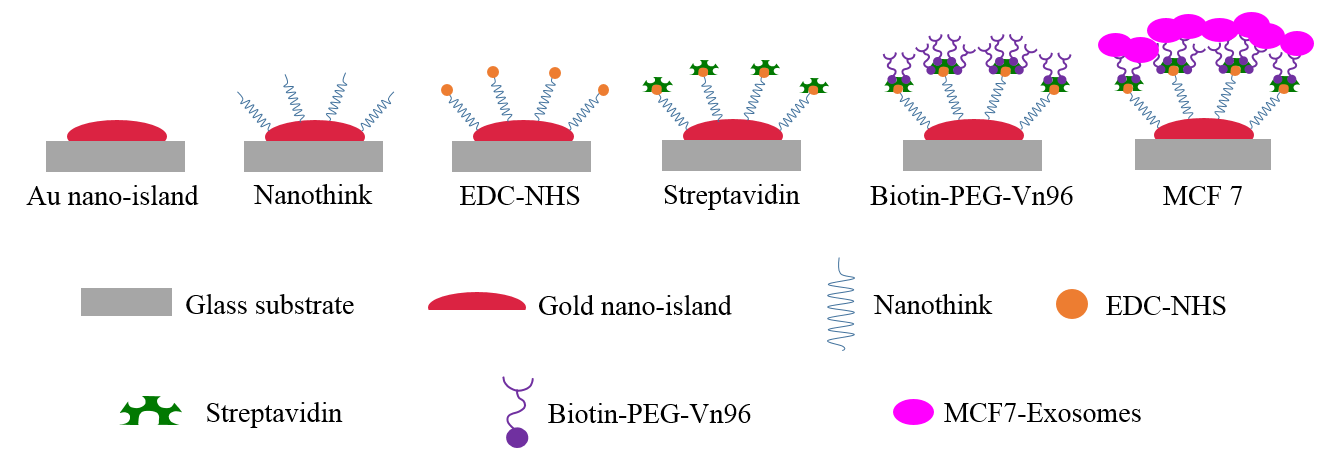


A

**Δλ = 4nm**

B

**Fig.S5. Biosensing protocol and their corresponding absorbance bands** (**A**) Schematic of the bio-sensing protocol (**B**) Au nano-island plasmon band corresponding to the different steps of the protocol

**Table S1. Concentrations and volume of the entities used in the biosensing protocol with their corresponding average LSPR shift**

| **Entity** | **Optimized concentration** | **Avg. Δλ (nm)** |
| --- | --- | --- |
| 11-MUA* | 5mM | 6 |
| EDC + NHS | 0.1M+0.05M | 4.8 |
| Streptavidin | 0.19 nM | 3.1 |
| Biotin-PEG-Vn96 | 0.87 nM | 5.7 |
| Exosomes (MCF 7) | 2%, 4%, 10%, 20%, 100% | - |

*11- Mercaptoundecanoic acid (the samples are immersed in the solution)
